# Trends in the use of antimuscarinics and alpha-adrenergic blockers in women with lower urinary tract symptoms in Taiwan: A nationwide, population-based study, 2007-2012

**DOI:** 10.1371/journal.pone.0220615

**Published:** 2019-10-07

**Authors:** Yu-Hua Lin, Wei-Yi Huang, Chi-Chih Chang, Yu-Fen Chen, Ling-Ying Wu, Hong-Chiang Chang, Kuo-How Huang

**Affiliations:** 1 Divisions of Urology, Department of Surgery, Cardinal Tien Hospital, Taipei, Taiwan; 2 Department of Chemistry, Fu Jen Catholic University, New Taipei City, Taiwan; 3 Graduate Institute of Biochemical and Pharmaceutical Science, Fu Jen Catholic University, New Taipei City, Taiwan; 4 Department of Healthcare and Medical Care, Veterans Affairs Council, Taipei, Taiwan; 5 Institute of Health and Welfare Policy, National Yang-Ming University, Taipei, Taiwan; 6 The Interdisciplinary Nanoscience Centre, Aarhus University, Aarhus, Denmark; 7 Department of Nursing, Kang-Ning Junior College of Medical Care and Management, Taipei, Taiwan; 8 Graduate Institute of European Studies, Tamkang University, Taipei, Taiwan; 9 Department of Urology, National Taiwan University Hospital, Taipei, Taiwan; University Medical Center Utrecht, NETHERLANDS

## Abstract

**Background:**

We aim to examine the trend in the use of antimuscarinics and off-label alpha-adrenergic blockers for treatment of lower urinary tract symptoms (LUTS) in a Taiwanese Women Cohort between 2007 and 2012.

**Methods:**

This population-based National Health Insurance Research Database (NHIRD) was used to examine the trends in the use of antimuscarinics or off-label alpha-adrenergic blockers in Taiwan. A sample of 1,000,000 individuals randomly drawn from the whole population of 23 million individuals who were registered in the NHI in 2005. From 2007 through 2012, women aged over 18 years whose claim record contained prescriptions of either of the two drugs for treatment of any of the LUTS-related diagnoses were identified and analyzed. The annual usage of the two drug classes were calculated by defined daily dose (DDD).

**Results:**

From 2007–2012, there was a 0.80 fold (69676.8 to 125104.3) increase in DDD of antimuscarinics in our cohort. The overall healthcare seeking prevalence of LUTS was 7.33% in 2007 and 12.38% in 2012, in a rising trend. The prevalence of antimuscarinics-treated LUTS in our cohort increased from 2.53 in 2007 to 3.41 per 1000 women in 2012. The prevalence of LUTS treated by antimuscarinics increased especially for those older than 60 years during the study period.

**Conclusions:**

This 6-year observational study provided the epidemiologic information of clinically significant LUTS of Asian female population. Moreover, there was a rising trend in the use of antimuscarinics and off-label alpha-adrenergic blockers in the population-based cohort.

## Introduction

Lower urinary tract symptom (LUTS) and overactive bladder syndrome (OAB) are common and distressing conditions in adults [1]. LUTS comprise storage/OAB symptoms, voiding symptoms, and postmicturition symptoms [2]. ICS defined OAB in 2002 as urgency with or without urge incontinence, usually with frequency and nocturia without proven infection or pathology.

Previous studies reported wide range of LUTS prevalence from 15.8% to 46.2%. and OAB prevalence from 6.0% to 29.9% [1, 3–5]. The variations may be associated with different study designs, methods of data acquisition (interview, web survey or questionnaire administration), definition of LUTS and OAB, or target populations. Additionally, most these studies were cross-sectional. A multi-national study by questionnaire showed the prevalence of LUTS was around 16.2–25.1% in men and 12.6–23.7% in women [3]. A population-based study in 6 European nations showed the overall prevalence of OAB in individuals aged over 40 years was 16.6% [4]. A nationwide study based on National Overactive Bladder Evaluation (NOBLE) Program in the United States showed the prevalence of OAB was 16.0% in men and 16.9% in women over 18 years of age [5]. To date, few large-scale epidemiologic studies on LUTS and OAB were performed in Asian population [1, 3–8].

In addition to impact on life quality, medical costs for the treatment of LUTS and OAB was surprisingly high. The cost of OAB in the United States in 2000 was estimated to 12.02 billion UDS [9]; increased to 65.9 billion UDS in 2007 [10]. Above all, LUTS and OAB have been known to be more prevalent in older population of both genders, and the prevalence and expenditures of LUTS and OAB are expected to increase by years due to aging societies in developed countries.

Anticholinergic agents (antimuscarinics) has been well-established as OAB treatment. Antimuscarinics suppressed involuntary bladder contraction and increase bladder capacity. The antimuscarinics used in market of Taiwan’s National Health Insurance (NHI) included oxybutynin, tolterodine, solifenacin, oxybutynin, trospium, propiverine. In addition, alpha1-adrenergic blocker, exhibiting the action to decrease smooth muscle tone of bladder outlet, were used for treatment of women with LUTS [11]. Only a few clinical trials showed the efficacy of alpha1-adrenergic blockers in voiding dysfunction of women without sufficient scientific evidence [12–14]; moreover, most adequately powered large studies ended up negative. The prescriptions of alpha1-adrenergic blockers for treatment of women with LUTS are off-label use.

In this study, we analyzed LUTS and usage of antimuscarinics or alpha-adrenergic blockers for treatment of LUTS in a nationwide, population-based female cohort in Taiwan.

## Materials and methods

This study utilized a dataset, the Longitudinal Health Insurance Database (LHID) 2005, collected from Taiwan’s National Health Insurance Data (NHIRD). LHID 2005 contains all the original claim data of 1,000,000 beneficiaries enrolled in year 2005 randomly sampled from the year 2005 Registry for Beneficiaries of the NHIRD. There are approximately 25.68 million individuals in this registry. There was no significant difference in the gender distribution (χ^2^ = 0.008, df = 1, p-value = 0.931) between the patients in the LHID2005 and the original NHIRD [15]. LHID2005 provided a good representation of the whole population of Taiwan.

Taiwan’s national health insurance program also provided the data on prescribed drugs, including substance, brand name, formulation, and dosage, expenditure and reimbursement the date of prescription and dispensing. This study was approved by the institutional review board of National Taiwan University Hospital (No.201306091W). The database used in this study were de-identified; therefore, the institutional review board waived informed consent from the enrolled patients.

### LUTS

LUTS diagnosis codes were defined by the International Classification of Diseases, Ninth Revision, Clinical Modification (ICD-9-CM) as follows: Storage symptoms included frequency (7884, 78841), polyuria (788.4, 788.42), incontinence (788.3, 788.30–788.39, 625.6,), nocturia (788.43) bladder hypertonicity (596.51), nocturnal enuresis (788.36), nocturia (788.43) and urinary urgency (788.63); Voiding symptoms included codes of voiding difficulty (788.1), urinary retention (788.2, 788.20), incomplete emptying (788.21), weak urine stream (788.6), post-void dribbling (788.35). splitting of urinary stream (788.61), slowing of urinary stream (788.62).

### Study sample

We first identified women aged over 18 years whose claim records included any record of using each of the two drugs. In the subgroup of alpha-adrenergic blockers usage, we excluded those who had the prescriptions doxazosin or terazosin with the diagnoses of hypertension and without the diagnoses of LTUS. In the subgroup of antimuscarinics usage, subjects who received the prescriptions of antimuscarinics, and had any codes of urinary tract infection (590.1, 590.2, 590.8, 590.9, 595.0, 595.9, 599.0, 996.64) were excluded from the sample. The defined daily dose (DDD) is a statistical measure of drug consumption, defined by the World Health Organization (WHO). The DDD is the calculated average maintenance dose per day for a drug when used for the main indications in adults. It is used to standardize the comparison of drug usage between different drugs or between different healthcare environments. DDD is the assumed average maintenance dose per day for a drug used for its main indication in adults. The DDD of five antimuscarinics drugs in this study were as follows: tolterodine 4 mg, solifenacin 5 mg, propiverine 30 mg, trospium 40 mg and oxybutynin 15 mg. Similarly, the DDD of alpha-adrenergic blockers were as follows: doxazosin 4mg, terazosin 10mg, alfuzosin 10mg, silodosin 8mg, and tamsulosin 0.4mg. We then analyzed DDDs of antimuscarinics or alpha-adrenergic blockers in 2007–2012.

Then we identified subjects who had any of specific diagnoses of LUTS and analyzed the prevalence of LUTS in Taiwan. We then calculated the prevalence of LUTS stratified by age categories and gender year by year during the study period.

### Statistical analysis

Age was classified into five categories: 18–39, 40–49, 50–59, 60–69, and ≥ 70 years. Linear regression analysis was used to evaluate trend in DDD of antimuscarinics or alpha-blockers prescriptions. LUTS, and newly-diagnosed rate for LUTS. All statistical analyses were performed using Excel of Microsoft Office 365.

## Results

**Fig 1:**
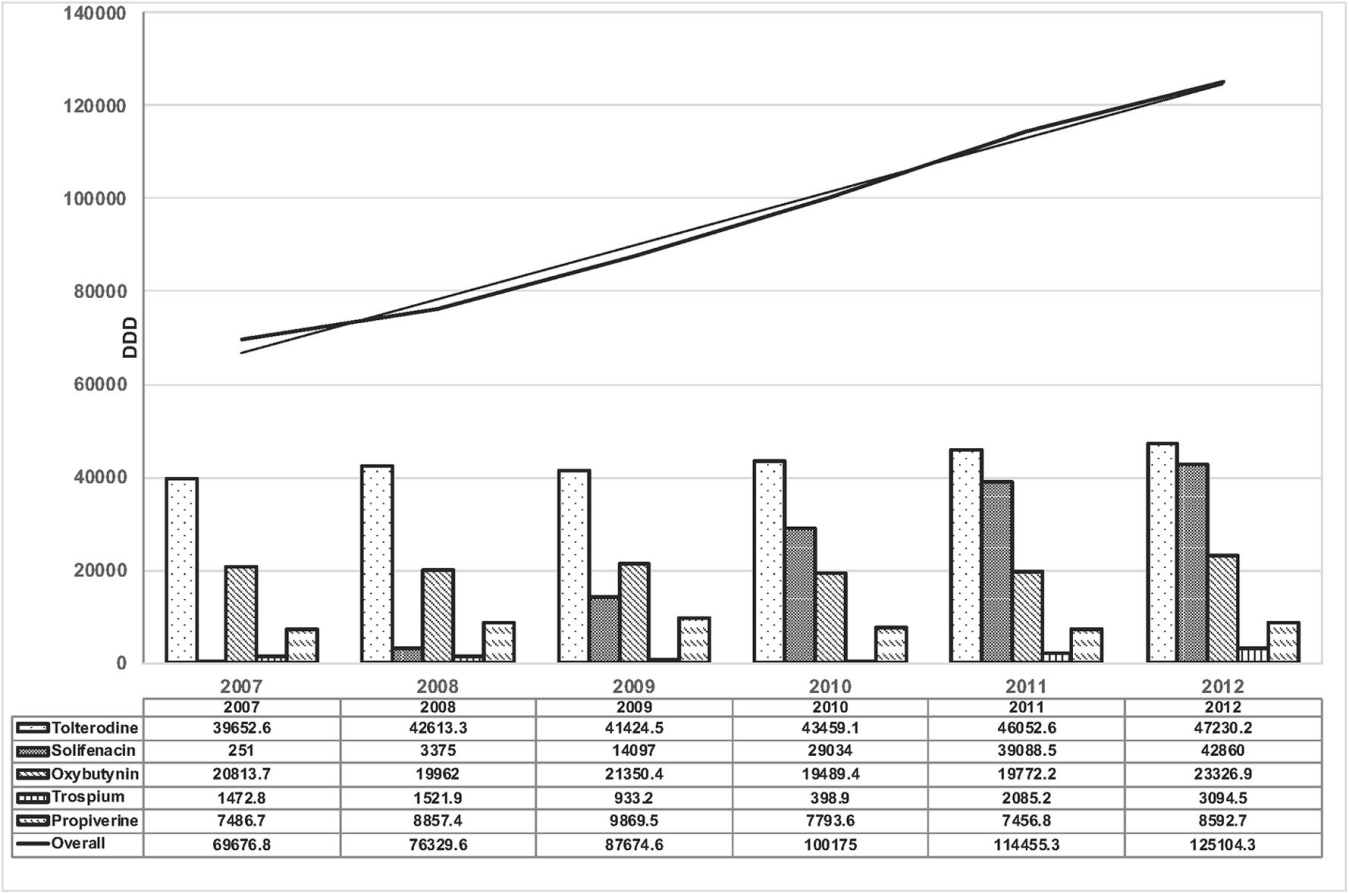
Annual trend in prescriptions of antimuscarinics calculated by defined daily dose (DDD) for the treatment of LUTS in women, 2007–2012. The black line is the overall DDD of antimuscarinics for each year. The dotted line is the trendline of linear regression test.

The annual DDDs of prescribed antimuscarinics for women with LUTS from 2007 through 2012 are shown in Fig 1. There was a 0.80 folds (69676.8 to 125104.3) increase in DDD of antimuscarinics in our cohort. Tolterodine was the mainly dispensed anticholinergic drug. Solifenacin markedly increased the DDDs by year after market introduction in 2007; whereas the prescriptions of oxybutynin and propiverine remained relatively steady during the 6-year study period.

The prescriptions of antimuscarinics were mainly in elderly. The DDD of prescribed antimuscarinics for women aged over 70 years accounted for 56% of the total usage of antimuscarinics.

**Fig 2:**
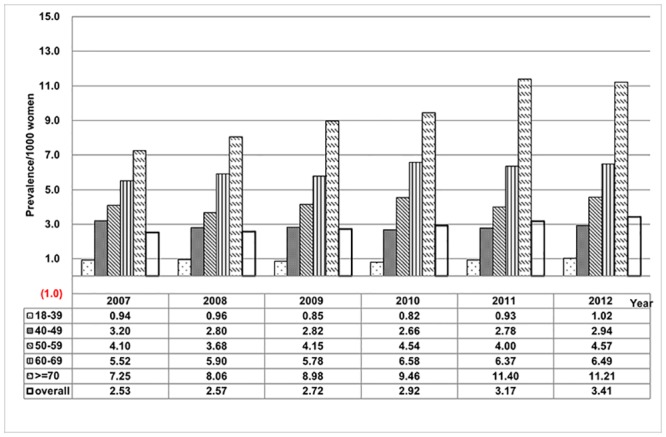
The age-stratified overall DDDs of antimuscarinics for treatment of female LUTS.

Annual prevalence for women with LUTS receiving antimuscarinics stratified by age are shown in Fig 2. The overall prevalence of antimuscarinics-treated LUTS increased from 2.53 in 2007 to 3.41 per 1000 women in 2012. The prevalence of LUTS treated by antimuscarinics increased especially for those older than 60 years.

**Fig 3:**
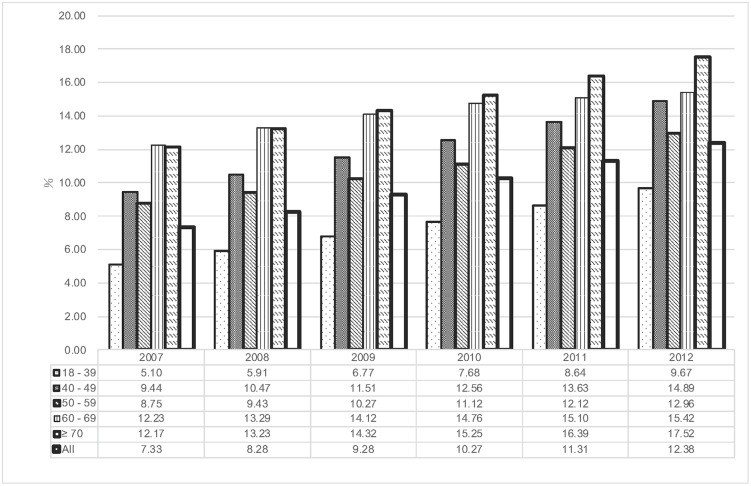
Overall and age-stratified prevalence for women receiving antimuscarinics for treatment of LUTS.

The number of subjects whose claim records contained LUTS diagnoses, the prescriptions of antimuscarinics or presriptions of alpha-adrenergic blockers categorized by age during the 6-year period are shown in supplement data (S1 Table). The prevalence of LUTS diagnoses categorized by age is shown in Fig 3. The overall healthcare seeking prevalence of LUTS was 7.33% in 2007 and 12.38% in 2012, in a rising trend. Consistently, the prevalence of LUTS were more prevalent at older age in our cohort.

The prescriptions of alpha-adrenergic blockers for treatment of LUTS in women remained off-label use due to insufficient scientific evidence. The results of annual DDDs of alpha-adrenergic blockers and age-specific prevalence of LUTS treated by alpha-adrenergic blockers are shown in supplement data (S1 and S2 Figs). Similarly, scientifically unproven off-label use of alpha-blockers for treatment of female LUTS increased during the 6-year analysis period in Taiwan.

## Discussion

An updated epidemiologic information of LUTS is important to improve the establishment of clinical guideline and health policy especially within the limited healthcare resources [16]. The majority of previous studies on the epidemiology of LUTS were mainly based on the data collected from self-completed questionnaire, personal interview, telephone interview, postal survey or web survey. It is noteworthy that high proportion of individuals with LUTS or OAB would not seek medical care or did not receive treatment. In large-scale study included 16,776 interviews in the six European countries, 60% of respondents with symptoms had consulted a doctor but only 27% were currently receiving treatment [4]. Chen et al. also reported similar results that only 27.1% of women with urinary incontinence sought medical help [6]. Our study is a population-based and nationwide study based on the medical claim records of Taiwan’s national health insurance research database (NHIRD). Our results demonstrated the prevalence of healthcare seeking LUTS and medications-treated LUTS, which were reasonably lower than the results of previous studies conducted by questionnaire or interview. In addition, in contrast to the cross-sectional design of previous studies, our study offered a more comprehensive picture of LUTS epidemiology by longitudinally observational design.

An epidemiologic study on LUTS in Taiwan based on NHIRD database has been conducted. The healthcare seeking prevalence of LUTS was 23% in 2000 and 38.4% in 2009 [8]. Our study showed the healthcare seeking prevalence of LUTS was rose from 7.33% in 2007 to 12.38% in 2012 among the Taiwanese female population. Our results showed a surprisingly high proportion, over 90%of subjects with LUTS did not receive medications for treatment. The possible reasons may include the inadequate physicians’ or patients’ perception of LUTS as a condition needing treatment and the symptoms is not bothersome or clinically-significant for medical treatment. The physicians and patients should be educated about the impact of LUTS or OAB on quality of life and the proper indications of medical treatment.

Most of previous studies indicated the prevalence of LUTS and OAB increased with age, especially those ≥ 60 year of age [1, 2, 5, 8–10, 16–21]. Our study also showed that prominently increased consumptions of antimuscarinics and alpha-adrenergic blockers were both found in elderly. The consumption of new anticholinergic agents, such as tolterodine and solifenacin, increased significantly; this may be related to marketing promotion, better compliance due to less side effects, or convenient dosing interval. Yet the prescription rates of oxybutynin remained stationary over the study period. Similarly, the increase of tamsulosin use by year may be related to a search for more selective drugs in health care providers.

There are several advantages in the present study. First, previous studies were mostly conducted in western countries in a cross-sectional design. Our study provided the 6-year observation of healthcare seeking prevalence of LUTS in Asian cohort. Second, the NHI covers over 99% of Taiwan’s 23-million population and the NHI databases are very representative of the general population. Moreover, NHIRD datasets are not designed for research purpose and can be more likely to be free of patient selection bias. Third, the NHIRD database included detailed prescription records. Each reimbursed outpatient or inpatient prescription are maintained in NHIRD to meet the need of pharmaco-epidemiological studies. In addition, the insurance claim data inevitably included some diagnostic errors. However, all claim records of the NHIRD are subject to a quality control process that included cross-comparison with medical chart information. In this way, some diagnostic errors in the raw claims data are rectified during that quality-control process. In this study, we simultaneously analyzed the subjects with the LUTS diagnostic codes and those who received treatment of two main drugs (anticholinergics and alpha blockers) for the treatment of LUTS. Strictly speaking, the prevalence of LUTS in this study was healthcare-seeking prevalence. Taiwan’s national health insurance program adopted strict and consistent criteria on prescription of antimuscarinics drugs for treatment of OAB. The patient should have one of the following two conditions: (1) voiding exceeds eight times per day; and (2) frequent urgency with or without urge incontinence; then healthcare providers was allowed to prescribe antimuscarinics. These criteria help to recognize study subjects of clinically significant storage symptoms of OAB. Nevertheless, the alpha-adrenergic blockers have been used as off-label treatment for female voiding dysfunction [12–14]. There are no established clinical guideline or regulations on the use of alpha-blockers for female voiding dysfunction in Taiwan. In addition, we found that most patients in Taiwan receiving tamsulosin treatment for female LUTS with the dose of 0.2mg rather than 0.4mg of DDD defined by WHO. In this study, we used DDD to examine the changes in drug utilization for treatment of LTUS over time, which can be used for international comparison. It is noteworthy that most patients prescribed an anti-muscarinic do not stay on treatment; calculation of DDD will systematically under-estimate use of these drugs.

## Conclusion

This nationwide population-based study demonstrated the 6-year observational study on prevalence of clinically significant LUTS treated by antimuscarinics or off-label alpha-adrenergic blockers in the Taiwanese women cohort. These findings provide valuable information of OAB and LUTS treatment and are helpful for care-providers and health insurance policy-makers.

## Supporting information

S1 Fig Annual trend in prescriptions of alpha-adrenergic blockers calculated by defined daily dose (DDD) for the treatment of LUTS in women, 2007–2012. The black line is the overall DDD of alpha-adrenergic blockers for each year. The dotted line is the trendline of linear regression test.
(TIFF)

S2 Fig The age-stratified overall DDDs of alpha-adrenergic blockers for treatment of female LUTS. (TIFF)

S1 Table (DOCX)
