# Supplementary figures and images for: Trends in the use of antimuscarinics and alpha-adrenergic blockers in women with lower urinary tract symptoms in Taiwan: A nationwide, population-based study, 2007-2012
Source: PLoS One. 2019 Oct 7;14(10):e0220615. doi: 10.1371/journal.pone.0220615 (PMC6779229; doi:10.1371/journal.pone.0220615)

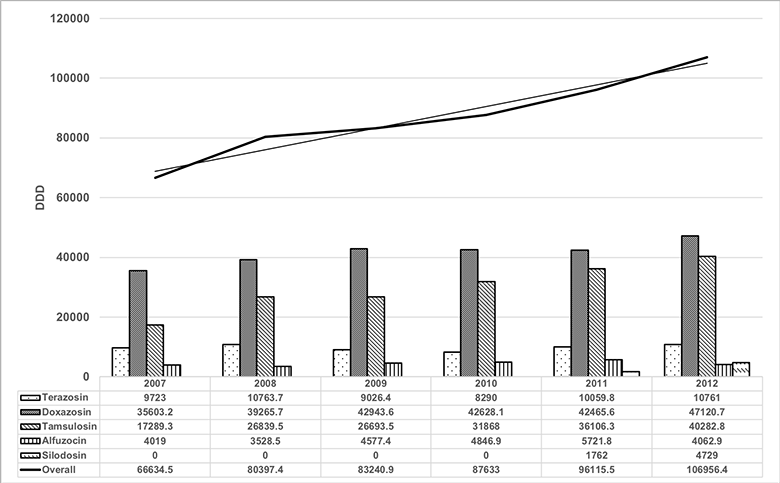

Supplement: S1 Fig — The black line is the overall DDD of alpha-adrenergic blockers for each year. The dotted line is the trendline of linear regression test. (TIFF) [file pone.0220615.s001.tiff]

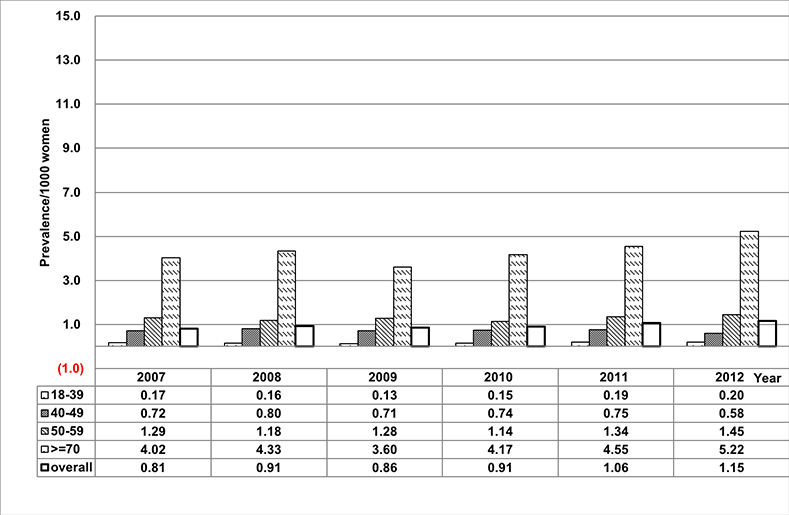

Supplement: S2 Fig — (TIFF) [file pone.0220615.s002.tiff]
