# Supplementary material for: Trends in the use of antimuscarinics and alpha-adrenergic blockers in women with lower urinary tract symptoms in Taiwan: A nationwide, population-based study, 2007-2012
Source: PLoS One. 2019 Oct 7;14(10):e0220615. doi: 10.1371/journal.pone.0220615 (PMC6779229; doi:10.1371/journal.pone.0220615)
Supplement: S1 Table — (DOCX) [file pone.0220615.s003.docx]

**S1: Table Number of subjects whose claim records contained the diagnoses of LUTS, the presciptions of anticholinergics or alpha-blockers categorized by age during 6-year study period, 2007-2012.**

|  | 18 - 39 | | 40 - 49 | 50 - 59 | | | 60 - 69 | | ≥ 70 | | All | | |  |
| --- | --- | --- | --- | --- | --- | --- | --- | --- | --- | --- | --- | --- | --- | --- |
| **Population** |  | |  |  | | |  | |  | |  | | |  |
| 2007 | 274486 | | 80982 | 65100 | | | 35147 | | 36565 | | 492280 | | |  |
| 2008 | 263810 | | 80938 | 67903 | | | 36076 | | 38317 | | 487044 | | |  |
| 2009 | 252646 | | 80837 | 70186 | | | 37524 | | 39997 | | 481190 | | |  |
| 2010 | 242656 | | 80724 | 72075 | | | 39370 | | 41757 | | 476582 | | |  |
| 2011 | 233577 | | 80143 | 73187 | | | 42214 | | 43320 | | 472441 | | |  |
| 2012 | 224537 | | 79009 | 74577 | | | 44969 | | 44791 | | 467883 | | |  |
| **Subjects with LUTS diagnosis and treated with anticholinergics** | | | | | | | | | | | | | | |
| **2007** | 259 | | 259 | 267 | | | 194 | | 265 | | 1244 | | |  |
| **2008** | 253 | | 227 | 250 | | | 213 | | 309 | | 1252 | | |  |
| **2009** | 215 | | 228 | 291 | | | 217 | | 359 | | 1310 | | |  |
| **2010** | 198 | | 215 | 327 | | | 259 | | 395 | | 1394 | | |  |
| **2011** | 218 | | 223 | 293 | | | 269 | | 494 | | 1497 | | |  |
| **2012** | 230 | | 232 | 341 | | | 292 | | 502 | | 1597 | | |  |
| **Subjects with LUTS diagnosis and treated with a-blockers** | | | | | | | | | | | | | | |
| **2007** | 46 | | 58 | 84 | | | 65 | | 147 | | 400 | | |  |
| **2008** | 41 | | 65 | 80 | | | 92 | | 166 | | 444 | | |  |
| **2009** | 34 | | 57 | 90 | | | 90 | | 144 | | 415 | | |  |
| **2010** | 37 | | 60 | 82 | | | 80 | | 174 | | 433 | | |  |
| **2011** | 45 | | 60 | 98 | | | 102 | | 197 | | 502 | | |  |
| **2012** | 46 | | 46 | 108 | | | 103 | | 234 | | 537 | | |  |
| **Subjects with LUTS** | | | | | | | | | | | | | | |
| **2007** | | 14,006 | 7,645 | | 5,699 | | | 4,298 | | 4,450 | | 36,098 |  |  |
| **2008** | | 15,582 | 8,477 | | 6,401 | | | 4,796 | | 5,071 | | 40,327 |  |  |
| **2009** | | 17,100 | 9,308 | | 7,206 | | | 5,298 | | 5,729 | | 44,641 |  |  |
| **2010** | | 18,626 | 10,138 | | 8,012 | | | 5,812 | | 6,367 | | 48,955 |  |  |
| **2011** | | 20,191 | 10,922 | | 8,867 | | | 6,375 | | 7,099 | | 53,454 |  |  |
| **2012** | | 21,705 | 11,762 | | 9,666 | | | 6,933 | | 7,848 | | 57,914 |  |  |
| **Subjects with storage LUTS** | | | | | | | | | | | | |  |  |
| **2007** | | 13,009 | 7,106 | | | 5,196 | | 3,810 | | 3,430 | | 32,551 |  |  |
| **2008** | | 14,479 | 7,871 | | | 5,837 | | 4,248 | | 3,869 | | 36,304 |  |  |
| **2009** | | 15,905 | 8,645 | | | 6,577 | | 4,674 | | 4,355 | | 40,156 |  |  |
| **2010** | | 17,329 | 9,413 | | | 7,299 | | 5,133 | | 5,817 | | 44,991 |  |  |
| **2011** | | 18,801 | 10,128 | | | 8,066 | | 5,597 | | 5,312 | | 47,904 |  |  |
| **2012** | | 20,214 | 10,905 | | | 8,777 | | 6,079 | | 5,828 | | 51,803 |  |  |
| **Subjects with voiding LUTS** | | | | | | | | | | | | |  |  |
| **2007** | | 1,429 | 975 | | | 885 | | 935 | | 1,587 | | 5,811 |  |  |
| **2008** | | 1,563 | 1,070 | | | 977 | | 1,022 | | 1,821 | | 6,453 |  |  |
| **2009** | | 1,676 | 1,141 | | | 1,077 | | 1,120 | | 2,045 | | 7,059 |  |  |
| **2010** | | 1,794 | 1,234 | | | 1,185 | | 1,200 | | 2,258 | | 7,671 |  |  |
| **2011** | | 1,910 | 1,313 | | | 1,279 | | 1,319 | | 2,547 | | 8,368 |  |  |
| **2012** | | 2,023 | 1,388 | | | 1,386 | | 1,410 | | 2,806 | | 9,013 |  |  |
